# Efficacy and safety of geptanolimab (GB226) for relapsed or refractory peripheral T cell lymphoma: an open-label phase 2 study (Gxplore-002)

**DOI:** 10.1186/s13045-021-01033-1

**Published:** 2021-01-12

**Authors:** Yuankai Shi, Jianqiu Wu, Zhen Wang, Liling Zhang, Zhao Wang, Mingzhi Zhang, Hong Cen, Zhigang Peng, Yufu Li, Lei Fan, Ye Guo, Liping Ma, Jie Cui, Yuhuan Gao, Haiyan Yang, Hongyu Zhang, Lin Wang, Weihua Zhang, Huilai Zhang, Liping Xie, Ming Jiang, Hui Zhou, Yuerong Shuang, Hang Su, Xiaoyan Ke, Chuan Jin, Xin Du, Xin Du, Li Liu, Yaming Xi, Zheng Ge, Ru Feng, Yang Zhang, Shengyu Zhou, Fan Xie, Qian Wang

**Affiliations:** 1 Department of Medical Oncology, National Cancer Center/National Clinical Research Center for Cancer/Cancer Hospital, Chinese Academy of Medical Sciences & Peking Union Medical College, Beijing Key Laboratory of Clinical Study on Anticancer Molecular Targeted Drugs, Beijing, China; 2 Department of Oncology, Jiangsu Cancer Hospital, Nanjing, China; 3 Department of Oncology, Linyi Cancer Hospital, Linyi, China; 4 Department of Lymphoma, Cancer Center, Union Hospital, Tongji Medical College, Huazhong University of Science and Technology, Wuhan, China; 5 Department of Hematology, Beijing Friendship Hospital, Capital Medical University, Beijing, China; 6 Department of Oncology, The First Affiliated Hospital of Zhengzhou University, Zhengzhou, China; 7 Department of Hematology, Lymphoma and Pediatric Oncology, Guangxi Medical University Affiliated Tumor Hospital and Oncology Medical College, Nanning, China; 8 Department of Oncology, The First Affiliated Hospital of Guangxi Medical University, Nanning, China; 9 Department of Hematology, Henan Cancer Hospital, Zhengzhou, China; 10 Department of Hematology, Jiangsu Province Hospital, Nanjing, China; 11 Department of Oncology, Shanghai East Hospital, Shanghai, China; 12 Department of Hematology, Sun Yat-Sen Memorial Hospital Sun Yat-Sen University, Guangzhou, China; 13 Department of Hematology, Gansu Provincial Cancer Hospital, Lanzhou, China; 14 Department of Hematology, The Fourth Hospital of Hebei Medical University, Shijiazhuang, China; 15 Department of Lymphoma, Zhejiang Cancer Hospital, Hangzhou, China; 16 Department of Oncology, The Fifth Affiliated Hospital Sun Yat-Sen University, Zhuhai, China; 17 Department of Oncology, Hainan General Hospital, Haikou, China; 18 Department of Hematology, The First Affiliated Hospital of Shanxi Medical University, Taiyuan, China; 19 Department of Lymphoma, Tianjin Medical University Cancer Institute and Hospital, Tianjin, China; 20 Department of Hematology, West China Hospital, Sichuan University, Chengdu, China; 21 Department of Oncology, West China Hospital, Sichuan University, Chengdu, China; 22 Department of Lymphoma and Hematology, Hunan Cancer Hospital, Changsha, China; 23 Department of Lymphoma Hematology and Oncology, Jiangxi Cancer Hospital, Nanchang, China; 24 Department of Lymphoma, The Fifth Medical Center of PLA General Hospital, Beijing, China; 25 Department of Hematology, Peking University Third Hospital, Beijing, China; 26 Department of Internal Medicine Section 4, Cancer Center of Guangzhou Medical University, Guangzhou, China; 27 Department of Hematology, Guangdong Provincial People’s Hospital, Guangdong Academy of Medical Sciences, Guangzhou, China; 28 Department of Hematology, Shenzhen Second People’s Hospital, Shenzhen, China; 29 Department of Hematology, Tangdu Hospital of the Fourth Military Medical University, Xi’an, China; 30 Department of Hematology, The First Hospital of Lanzhou University, Lanzhou, China; 31 Department of Hematology, Zhongda Hospital Southeast University, Nanjing, China; 32 Department of Hematology, Nanfang Hospital, Southern Medical University, Guangzhou, China; 33 Department of Oncology, The Second Hospital of Dalian Medical University, Dalian, China; 34 Department of Medical Science, Genor Biopharma Co., Ltd., Shanghai, China

**Keywords:** T cell lymphomas, PD-1 inhibitor, Immunotherapy

## Abstract

**Background:**

Peripheral T cell lymphoma (PTCL) is a rare disease and recent approved drugs for relapsed/refractory (r/r) PTCL provided limited clinical benefit. We conducted this study to evaluate the efficacy and safety of geptanolimab (GB226), an anti-PD-1 antibody, in r/r PTCL patients.

**Methods:**

We did this single-arm, multicenter phase 2 study across 41 sites in China. Eligible patients with r/r PTCL received geptanolimab 3 mg/kg intravenously every 2 weeks until disease progression or intolerable toxicity. All patients who received at least one dose of geptanolimab and histological confirmed PTCL entered full analysis set (FAS). The primary endpoint was objective response rate (ORR) in FAS assessed by the independent radiological review committee (IRRC) per Lugano 2014 criteria.

**Results:**

Between July 12, 2018, and August 15, 2019, 102 patients were enrolled and received at least one dose of geptanolimab. At the data cutoff date (August 15, 2020), the median follow-up was 4.06 (range 0.30–22.9) months. For 89 patients in FAS, 36 achieved objective response (40.4%, 95% CI 30.2–51.4), of which 13 (14.6%) were complete response and 23 (25.8%) had partial response assessed by IRRC. The median duration of response (DOR) was 11.4 (95% CI 4.8 to not reached) months per IRRC. Patients with PD-L1 expression ≥ 50% derived more benefit from geptanolimab treatment compared to < 50% ones (ORR, 53.3% vs. 25.0%, *p* = 0.013; median PFS 6.2 vs. 1.5 months, *p* = 0.002). Grade ≥ 3 treatment-related adverse events occurred in 26 (25.5%) patients, and the most commonly observed were lymphocyte count decreased (*n* = 4) and platelet count decreased (*n* = 3). Serious adverse events were observed in 45 (44.1%) patients and 19 (18.6%) were treatment related.

**Conclusions:**

In this study, geptanolimab showed promising activity and manageable safety profile in patients with r/r PTCL. Anti-PD-1 antibody could be a new treatment approach for this patient population.

*Trial registration*: This clinical trial was registered at the ClinicalTrials.gov (NCT03502629) on April 18, 2018.

## Introduction

According to the 2016 World Health Organization classification of lymphoid neoplasms [1], peripheral T cell lymphomas (PTCLs) were classified into 27 distinct subtypes. This heterogeneous disease entity accounts for around 25% of non-Hodgkin lymphoma (NHL) patients in China and about 10% in USA and Europe [2, 3]. Due to its heterogeneity and rarity, most of the clinical studies conducted in PTCL were retrospective with a small sample size in which the treatment regimens largely followed B cell NHL. However, worse prognosis was associated with PTCLs when compared to B cell NHL as 5-year overall survival (OS) rate was only 32% in most common PTCL subtypes (PTCL not otherwise specified [PTCL-NOS], angioimmunoblastic T cell lymphoma [AITL] and natural killer [NK]/T cell lymphoma) [3].

Anthracycline-containing regimens, for example CHOP (cyclophosphamide, doxorubicin, vincristine, prednisone) and CHOP plus etoposide (CHOEP), were commonly conducted in the first-line treatment of PTCL which have remained as the recommended approaches for most PTCL subtypes except for NK/T cell lymphoma in the first-line setting. For NK/T cell lymphoma, anthracycline-containing regimens usually provide poor clinical outcomes, whereas asparaginase-based chemotherapy, such as DDGP (dexamethasone, cisplatin, gemcitabline, and pegaspargase), can yield significant improvement in survival and better tolerability [4]. High-dose chemotherapy followed by autologous stem-cell transplantation (ASCT) has also been investigated [5–9]. For relapsed or refractory (r/r) PTCLs, novel agents like antifolate (pralatrexate) or histone deacetylase (HDAC) inhibitors (romidepsin, belinostat, vorinostat and chidamide) have shown an efficacy with objective response rates (ORRs) below 30% [10–16].

Brentuximab vedotin, a CD30-directed antibody–drug conjugate, was recently reported for a promising efficacy in CD30-expressing systemic r/r anaplastic large cell lymphoma (ALCL) with an ORR of 86% and duration of response (DOR) of 12.6 months [17]. Some evidence indicated that brentuximab vedotin could also bring clinical benefit to CD30-positive PTCL-NOS patients, not other subtypes of PTCL [18]. The unmet medical needs remain as new drugs for other targets to be explored and possibility to improve the treatment paradigm for r/r PTCL.

In the past several years, immune checkpoint inhibitor (ICI) has revolutionized the clinical management of cancer [19]. The activity of anti-programmed cell death protein-1 (PD-1) has been observed in r/r classic Hodgkin lymphoma and primary mediastinal large B cell lymphoma, in which programmed death-ligand 1 (PD-L1) upregulation is commonly seen [20–24]. Nevertheless, in r/r diffuse large B cell lymphoma (DLBCL), nivolumab showed limited efficacy with an independently assessed ORR of 10% and 3% in the ASCT-failed cohort and ASCT-ineligible cohort, respectively [25].

For T cell lymphoma, anti-PD-1 antibodies have shown modest efficacy in r/r PTCL, but results were limited by a small sample size and concerns of rapid disease progression [26, 27]. Thus, a better understanding of anti-PD-1 treatment for r/r PTCL patients and identification of biomarkers for immunotherapy optimization are required.

Geptanolimab (GB226) is a recombinant anti-PD-1 humanized monoclonal antibody that selectively blocks the interaction of PD-1 with its ligands, manufactured by Genor Biopharma Co., Ltd (Shanghai, China). Based on the phase 1 study of geptanolimab in lymphoma and advanced solid tumors (data not published), 3 mg/kg every 2 weeks was selected as recommended. Herein, we present the results of this phase 2 study (Gxplore-002) to determine the efficacy and safety of geptanolimab in patients with r/r PTCL, and assess the possible correlation between PD-L1 expression and its clinical outcome (NCT03502629).

## Methods

### Patients

Eligible individuals were patients aged 18 years and older with histologically confirmed PTCL, who had failed at least one prior systemic therapy. Additional criteria of enrolment included at least one bidimensionally measurable lesion according to investigator assessment as defined by Lugano criteria [28], an Eastern Cooperative Oncology Group (ECOG) performance status (PS) of 0–1, life expectancy of at least 3 months and at least 4 weeks after ASCT or major operation. Patients were also required to have adequate organ functions: absolute neutrophil count ≥ 1.0 × 10^9^/L, platelet count ≥ 75 × 10^9^/L, hemoglobin ≥ 80 g/L, total bilirubin ≤ 1.5 × the upper limit of normal (ULN), alanine aminotransferase (ALT) and aspartate aminotransferase (AST) ≤ 2.5 × ULN (if liver involvements are present, ALT and AST up to 5 × ULN), serum creatinine ≤ 1.5 × ULN or creatinine clearance ≥ 50 mL/min (calculated by Cockcroft and Gault equation).

Patients were excluded if histologically classified as adult T cell leukemia/lymphoma (ATLL) or AITL; concomitant receipt of immunosuppressive therapy; prior exposure to any anti-PD-1/PD-L1 or anti-cytolytic T lymphocyte-associated antigen-4 (CTLA-4) antibody; treatment with systematic corticosteroids (> 10 mg daily prednisone equivalent) within 2 weeks; active or history of autoimmune disease except for type I diabetes, controllable hypothyroidism with replacement treatment, controllable skin disorders without systematic treatment, celiac disease within control; human immunodeficiency virus (HIV) infection, anti-treponema pallidum antibody (TP-Ab) positive or active hepatitis B or C; use of any investigational agent, biologics or devices within 30 days (or at least five periods of half-life) before study treatment. Pathological diagnosis was done by study site pathologists for study enrolment, and patient eligibility was determined by site investigators. Central pathology review was conducted retrospectively upon patient consent.

The study was done in accordance with the Declaration of Helsinki and International Council for Harmonisation (ICH) guidelines for Good Clinical Practice. The study protocol is available in Additional file 1. The protocol, protocol amendments and patient informed consent were reviewed and approved by the relevant independent ethics committee at each participating study site prior to implementation. Written informed consent was obtained from all patients prior to enrolment.

### Study design, treatment and assessment

We conducted this open-label, single-arm, phase 2 study across 41 sites in China to evaluate the activity and safety profile of geptanolimab in r/r PTCL. Eligible patient received geptanolimab intravenously at a dose of 3 mg/kg every 2 weeks until disease progression, death, unacceptable toxicity, withdrawal of consent or end of the study (i.e., a maximum treatment duration of 2 years of the last subject, termination of treatment, consent withdrawal, lost to follow-up or death, whichever occurs first). Geptanolimab treatment was permitted to continue beyond the first assessment of progressive disease (PD) if the patient could benefit from the treatment with acceptable toxicity assessed by the investigator. Dose modification was not allowed. Patients with dose interruptions for more than 4 weeks should permanently discontinue the treatment.

Treatment response was assessed every 6 weeks until week 48 and every 12 weeks thereafter during the treatment phase. Treatment response was assessed using computed tomography (CT) or magnetic resonance imaging (MRI) and complementary information from positron emissions tomography and computed tomography (PET-CT). PET-CT was performed at baseline, week 13 and termination of treatment. All radiographic records of enrolled patients were provided to independent radiological review committee (IRRC). Efficacy was assessed based on individual best objective response by both investigators and IRRC according to the Lugano criteria [28]. Survival follow-up was done every 3 months until patient death, lost to follow-up or the end of the study.

### Outcomes

The primary endpoint of this study was ORR, defined as the proportion of patients with a best overall response (including complete response [CR] and partial response [PR]) per the Lugano criteria and assessed by IRRC. The secondary outcomes included disease control rate (DCR), time to response (TTR), duration of response (DOR), PFS, OS, safety and immunogenicity of geptanolimab.

Safety was monitored for 30 days from the last dose of geptanolimab for all patients or before any anticancer therapy began. If no new anticancer treatment has commenced, the safety follow-up should be repeated as far as possible for  90 days after the last dose of geptanolimab. Adverse events (AEs) were assessed by National Cancer Institute Common Terminology Criteria for Adverse Event (NCI CTCAE), version 4.03 and classified as related, possibly related, unlikely related, unrelated and uncertainly related to treatment assessed by investigator. Treatment-related AEs (TRAEs) were those AEs labeled related, possibly related, or uncertainly related to study drug. As an ICI, geptanolimab could cause a spectrum of immune-related adverse events (irAEs), which is recognized and managed according to National Comprehensive Cancer Network guideline for Management of Immunotherapy-Related Toxicities [29].

### Statistical analysis

To provide roughly 85% power to reject the null hypothesis that the true proportion of patients achieving an objective response is 15% or fewer and given a one-sided alpha of 2.5%, 73 patients were required. Considering the possible drop-off rate of 15%, at least 86 patients were to be recruited.

All patients receiving at least one dose of geptanolimab were included in the safety analysis set. Patients with retrospectively histological confirmation per central pathology review (*n* = 89) entered the full analysis set (FAS). Efficacy analysis was performed in FAS and per protocol set (PPS), assessed by IRRC and by investigators. ORR was the proportion of patients with CR or PR as the best overall response. DCR was the percentage of patients achieving either a CR or PR or SD. We summarized AEs as the proportion of the total number of patients receiving at least one dose of geptanolimab.

Exact binominal confidence intervals (CIs) were calculated for response outcomes. PFS (from first geptanolimab administration to progression or death, whichever occurs first), OS (from first geptanolimab administration to death), DOR (from the date of first documented response to the date of first documented disease progression or death from any cause) and TTR (time from first geptanolimab administration to first documentation of response) were assessed using the Kaplan–Meier method. All statistical analyses were performed by using SAS version 9.4.

This trial was registered at the ClinicalTrials.gov (NCT03502629).

### Biomarker assessment

Immunogenicity was evaluated by the number of patients with antidrug antibody (ADA) positive. Serum samples were collected at baseline, week 1, 2, 6 and followed by every six weeks. An immunological ligand-binding assay (LBA) was developed and validated for the detection and quantitation of anti-geptanolimab antibodies in serum collected.

Archival tumor tissues were required from all patients prior to treatment. PD-L1 immunohistochemistry (IHC) was assessed on formalin-fixed paraffin-embedded (FFPE) tumor biopsy samples using Ventana PD-L1 (SP263; Roche) rabbit monoclonal primary antibody for exploratory analysis. Measure of PD-L1 expression built on the estimated percentage of PD-L1-stained cells. Genomic DNA was extracted from FFPE tumor samples using ReliaPrep FFPE gDNA Miniprep System (Promega) following the manufacturer’s instructions. Microsatellite instability (MSI) analysis using MSI Analysis System version 1.2 (Promega) and tumor mutation burden (TMB) assessed with hybridization capture of exons from 440 cancer-related genes were performed (Details in Additional file 1).

## Results

### Patients and treatment

**Fig. 1:**
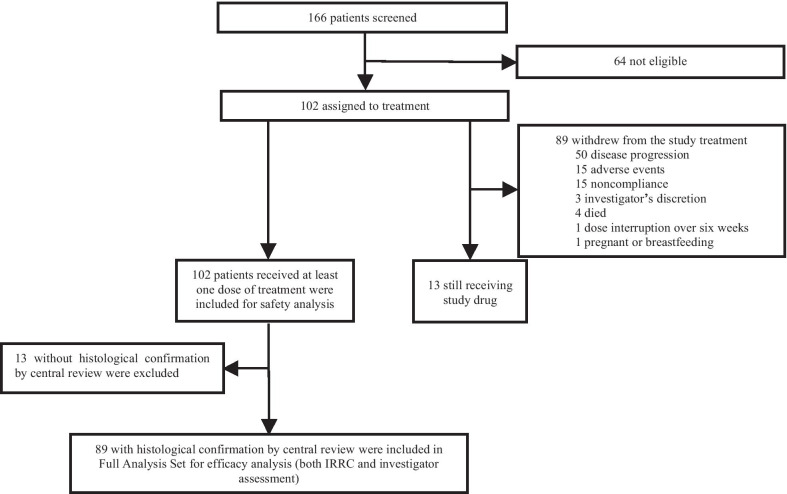
Patient disposition of all screened patients. IRRC, independent radiological review committee. *Note* Pathological diagnosis was done by study site pathologists for study enrolment and patient eligibility was determined by site investigators. Central pathology review was conducted retrospectively upon patient consent. Retrospectively pathological confirmation per central pathology review was not available in 13 patients, leaving 89 patients entering FAS

**Fig. 2:**
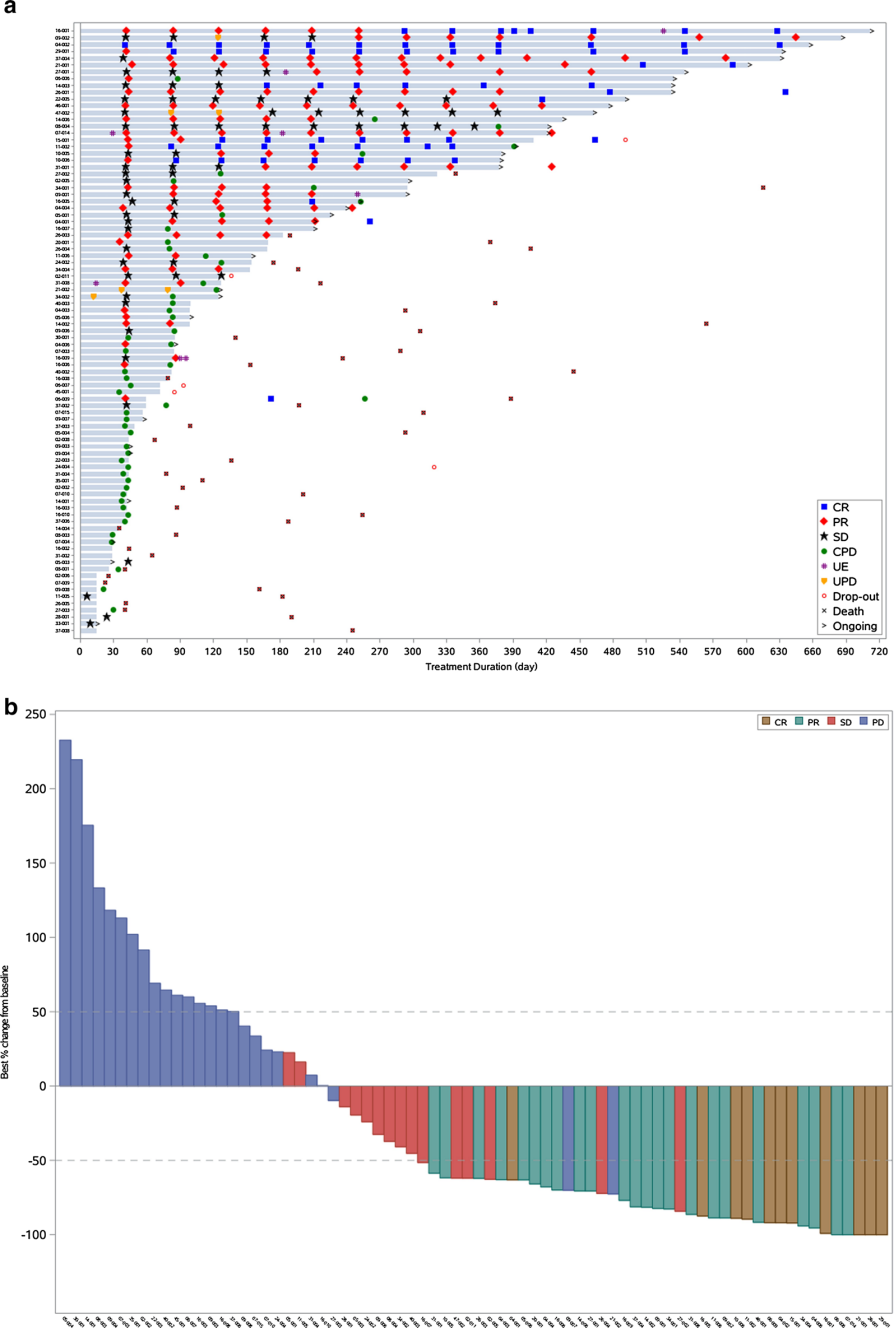
**a** Duration of treatment for full analysis set population (N = 89). **b** Best percentage change from baseline in target lesion size for full analysis set population based on an independent radiological review committee assessment (N = 74). CR, complete response; PR, partial response; SD, stable disease; CPD, confirmed progressive disease; UE, unevaluable; UPD, unconfirmed progressive disease; PD, progressive disease**.** *Note* Out of 89 patients in the full analysis set, five patients did not have measurable target lesion at baseline per central review, eight patients withdrew before first response assessment and two patients had disease progression on the basis of physical examination by investigators without radiographic record

A total of 102 patients were recruited in this study at 41 sites in China from July 12, 2018, to August 15, 2019 (Additional file 1: Table S1). The average time for enrolled patients after the initial diagnosis of PTCL was 0.94 years (standard deviation: 1.53), and 58.8% of them had two or more prior treatments. At study entry, 84 (82.4%) patients had advanced disease and 13 (12.7%) patients had bone marrow involvement at baseline. All patients had received prior systematic treatment. For patients diagnosed with extranodal NK/T cell lymphoma, nasal type (ENKTL), 21 (91.3%) out of 23 patients received prior asparaginase-based chemotherapy. For other subtypes of PTCL except for ENKTL, 77 (97.5%) out of 79 patients were previously treated with anthracycline-containing chemotherapy. Twenty-four (23.5%) patients had received chidamide; 7 (6.9%) had undergone ASCT. Baseline characteristics of the 102 patients are shown in Table 1. Collectively, 89 patients with retrospectively histological confirmation per central pathology review were included in FAS (Additional file 1: Table S2). Of them, the most common histological subtypes were PTCL-NOS (*n* = 28 [31.5%]), ENKTL (*n* = 19 [21.3%]), anaplastic lymphoma kinase-negative anaplastic large cell lymphoma (ALCL ALK-, *n* = 13 [14.6%]) and anaplastic lymphoma kinase-positive anaplastic large cell lymphoma (ALCL ALK+, *n* = 7 [7.9%]). At the cutoff date, 54 (52.9%) deaths were recorded and 13 (12.7%) patients were still under treatment (Fig. 2a). Disease progression was the most common reason for patient withdrawal, occurring in 50 (49.0%) patients. Eleven (10.8%) patients discontinued for TRAEs including two platelet count decreased, two autoimmune hemolytic anemia and two pneumonitis (Additional file 1: Table S3). All 102 patients were included in safety analysis and 89 with histological confirmation of PTCL were in FAS for efficacy analysis (Fig. 1).

**Table 1:** Patient baseline demographic and clinical characteristics (*N* = 102)

Patient characteristics	*N* (%)
Age	
Median age, years (range)	52.5 (18–78)
< 65	87 (85.3)
≥ 65	15 (14.7)
Gender	
Male	70 (68.6)
Female	32 (31.4)
ECOG PS	
0	19 (18.6)
1	83 (81.4)
Prior lines of systemic therapy	
1	42 (41.2)
2	34 (33.3)
3 or above	26 (25.5)
Stage of disease^#^	
I–II	17 (16.7)
III–IV	84 (82.4)
Pathological subtype^$^	
PTCL-NOS	41 (40.2)
ENKTL	23 (22.5)
ALCL ALK-	12 (11.8)
ALCL ALK+	7 (6.9)
Other subtypes*	19(18.6)
Prior therapies	
Multi-agent regimen	
For ENKTL	
Asparaginase-based chemotherapy^†^	21 (91.3)
For other subtypes of PTCL	
Anthracycline-containing chemotherapy^‡^	77 (97.5)
Single-agent regimen	
Chidamide	24 (23.5)
Gemcitabine	55 (53.9)
Methotrexate	10 (9.8)
Bortezomib	1 (1.0)
Radiotherapy	32 (31.4)
Autologous stem-cell transplantation	7 (6.9)

ENKTL, extranodal natural killer/T cell lymphoma, nasal type; ALCL, anaplastic large-cell lymphoma; ALK, anaplastic lymphoma kinase; CHOP, cyclophosphamide, doxorubicin, vincristine, prednisone; ECOG, Eastern Cooperative Oncology Group; PS, performance status; NOS, not otherwise specified; PTCL, peripheral T cell lymphoma

^#^Stage information of one case was not available

^$^PTCL was pathologically diagnosed by study site pathologist for study enrolment

^*^Other subtypes included: 11 cases of unclassifiable PTCL, 3 cases of type II enteropathy-associated T cell lymphoma (EATL, type II), 3 cases of mycosis fungoides (MF), 1 case of skin γ σ cutaneous T cell lymphomas (γ σ CTCL), and 1 case of primary cutaneous CD4 positive T cell lymphoma (CD4 + PCTCL)

^†^The proportion (%) was defined as the number of patients receiving asparaginase-based chemotherapy divided by the number of those diagnosed with ENKTL

^‡^The proportion (%) was defined as the number of patients receiving anthracycline-containing chemotherapy divided by the number of those diagnosed with other subtypes of PTCL except for ENKTL

### Efficacy

As of August 15, 2020, the median follow-up was 4.06 (range 0.30–22.9) months. Efficacy data in the total enrolled population (*n* = 102) and FAS (*n* = 89) are presented in Table 2, with no significant difference observed between the two sets. Of FAS population, 36 of 89 (40.4%, 95% CI, 30.2% to 51.4%) patients achieved an overall response by IRRC with a median TTR of 1.4 (95% CI, 1.4 to 1.5) months for responders; disease control was achieved in 53 (59.6%; 95% CI, 48.6% to 69.8%) patients. Assessed by investigators, 34 of 89 (38.2%, 95% CI, 28.1% to 49.1%) achieved an overall response (Table 2). The lower limit of the 95% CI (30.2%) of ORR by IRRC was above the prespecified threshold of response. Best reductions from baseline in tumor burden are shown in Fig. 2b.

**Table 2:** Efficacy of geptanolimab

Endpoint	Full analysis set		All patients enrolled	
	Investigator assessed (*n* = 89)	IRRC assessed (*n* = 89)	Investigator assessed (*n* = 102)	IRRC assessed (*n* = 102)
ORR				
No. of patients	34	36	37	39
% of patients (95% CI)	38.2 (28.1 to 49.1)	40.4 (30.2 to 51.4)	36.3 (27.0 to 46.4)	38.2 (28.8 to 48.4)
DCR				
No. of patients	49	53	54	57
% of patients (95% CI)	55.1 (44.1 to 65.6)	59.6 (48.6 to 69.8)	52.9 (42.8 to 62.9)	55.9 (45.7 to 65.7)
Objective response, *n* (%)				
CR	8 (9.0)	13 (14.6)	8 (7.8)	14 (13.7)
PR	26 (29.2)	23 (25.8)	29 (28.4)	25 (24.5)
SD	15 (16.9)	17 (19.1)	17 (16.7)	18 (17.6)
PD	32 (36.0) ^#^	28 (31.5)	37 (36.3)^&^	34 (33.3)
Not reported^§^	8 (9.0)	8 (9.0)	11 (10.8)	11 (10.8)
Median DOR, months				
Median (95% CI)	4.0 (1.5 to NR)	11.4 (4.8 to NR)	4.2 (1.5 to NR)	7.4 (5.1 to NR)
% of patients ≥ 6 months (95% CI)	46.9 (29.1 to 62.8)	60.5 (41.8 to 74.8)	48.6 (31.4 to 63.7)	60.9 (43.0 to 74.7)
% of patients ≥ 12 months (95% CI)	46.9 (29.1 to 62.8)	48.5 (29.6 to 65.0)	40.8 (23.9 to 57.0)	43.8 (26.3 to 60.0)

ORR, objective response rate; DCR, disease control rate; CR, complete response; PR, partial response; SD, stable disease; PD, progressive disease; IRRC, independent radiological review committee; DOR, duration of response; CI, confidence interval; NR, not reached

^#^21 patients had confirmed progressive disease; 11 patients with a first progressive disease and still under treatment

^&^23 patients had confirmed progressive disease; 14 patients with a first progressive disease and still under treatment

^§^Patients did not have post-baseline efficacy evaluation due to early termination or protocol violation

**Fig. 3:**
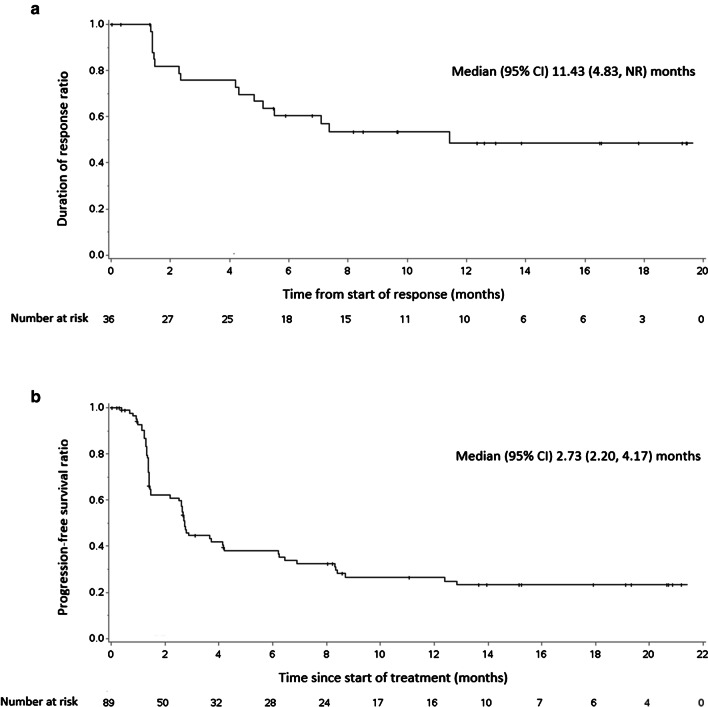
**a** Kaplan–Meier estimated duration of response in full analysis set population based on an independent radiological review committee (IRRC) assessment of patients (N = 36). **b** Kaplan–Meier estimated progression-free survival in full analysis set population based on an IRRC assessment (N = 89). NR, not reached; CI, confidence interval

Among the 36 patients who responded to geptanolimab, 13 (14.6%) patients achieved CR and 23 (25.8%) achieved PR. Among them, the majority of patients had an ongoing response with a median DOR of 11.4 (95% CI, 4.8 to not reached) months (Fig. 3a). The 12-month DOR was 48.5% (95% CI, 29.6% to 65.0%) (Table 2). Of FAS population, the median PFS was 2.7 (95% CI, 2.2 to 4.2; Fig. 3b) months with a range of 0 to 21.2 months, and the median OS was 14.6 (95% CI, 9.6 to not reached) months, with a range of 0.7 to 22.9 months. No significant difference was observed between efficacy result per IRRC and investigator (*p* = 0.356).

Subgroup analysis was performed on FAS population showing consistent response across different age, gender, clinical stage, baseline bone marrow involvement and prior ASCT. Elevated lactate dehydrogenase (LDH) level was adversely correlated with patient response (ORR, 49.0% vs. 28.9%, *p* = 0.081; DCR, 72.5% vs. 42.1%, *p* = 0.005) and OS (not reached vs 9.5 months, *p* = 0.012). Additionally, patients who were heavily treated (≥ 2 lines of systemic treatment) presented similar response (ORR 34.6% vs. 48.6%, *p* = 0.197) but shorter PFS (2.5 vs. 8.4 months, *p* = 0.001). Nevertheless, prior exposure to chidamide had no impact on patient response (*p* = 0.313). Among the 20 patients with previous failure of chidamide, response was observed in 6 patients (30.0%, 95% CI 11.9 to 54.3) (Additional file 1: Table S4). Among patients in FAS, the highest IRRC-assessed ORR was observed in ENKTL reaching 63.2% (12/19), followed by 53.8% (7/13) in ALCL ALK-, 42.9% (3/7) in ALCL ALK+ and 17.9% (5/28) in PTCL-NOS (Additional file 1: Table S5). Among the four patients classified as AITL according to the retrospectively central pathology review, two (50.0%) achieved PR and two (50.0%) achieved SD.

### Safety

For 102 safety-evaluable patients, 94 (92.2%) patients experienced at least one AE during the study and 35 (34.3%) patients only had mild (grade 1–2) AEs. The most common AEs included white blood cell count decreased (*n* = 36, 35.3%), anemia (*n* = 34, 33.3%), fever (*n* = 27, 26.5%), platelet count decreased (*n* = 24, 23.5%), neutrophil count decreased (*n* = 20, 19.6%), lymphocyte count decreased (*n* = 17, 16.7%), hypokalemia (*n* = 16, 15.7%), AST increased (*n* = 16, 15.7%), cough (*n* = 16, 15.7%), upper respiratory infection (*n* = 15, 14.7%), anorexia (*n* = 15, 14.7%), weight loss (*n* = 14, 13.7%), ALT increased (*n* = 13, 12.7%) and pruritus (*n* = 13, 12.7%). According to investigator’s assessment, TRAEs occurred in 80.4% of patients (*n* = 82; Table 3). TRAEs occurring in ≥ 10% were white blood cell count decreased (*n* = 20, 19.6%), fever (*n* = 14, 13.7%) and anemia (*n* = 13, 12.7%). A total of 26 (25.5%) patients occurred grade 3 or above TRAEs. The most commonly reported grade 3 or above TRAEs were lymphocyte count decreased (*n* = 4, 3.9%), platelet count decreased (*n* = 3, 2.9%), white blood cell count decreased (*n* = 2, 2.0%), anemia (*n* = 2, 2.0%), lung infection (*n* = 2, 2.0%), upper respiratory infection (*n* = 2, 2.0%), abnormal liver function (*n* = 2, 2.0%), infection (*n* = 2, 2.0%) and autoimmune hemolytic anemia (*n* = 2, 2.0%). Severe adverse events (SAEs) were reported in 45 (44.1%) patients, and 26 SAEs were judged to be treatment related in 19 (18.6%) patients; the most common treatment-related SAE were platelet count decreased (*n* = 3, 2.9%) and pneumonitis (*n* = 3, 2.9%). There was no death attributed to the geptanolimab per investigator assessment.

**Table 3:** Treatment-related adverse events

Treatment-related adverse events*	Grade 1–2	Grade 3	Grade 4	Grade 5
White blood cell count decreased	18 (17.6%)	1 (1.0%)	1 (1.0%)	0
Fever	13 (12.7%)	1 (1.0%)	0	0
Anemia	11 (10.8%)	2 (2.0%)	0	0
Lymphocyte count decreased	6 (5.9%)	3 (2.9%)	1 (1.0%)	0
Neutrophil count decreased	9 (8.8%)	0	1 (1.0%)	0
Pruritus	8 (7.8%)	1 (1.0%)	0	0
Platelet count decreased	6 (5.9%)	1 (1.0%)	2 (2.0%)	0
Pneumonitis	6 (5.9%)	0	1 (1.0%)	0
Rash	4 (3.9%)	1 (1.0%)	0	0
Abnormal liver function	3 (2.9%)	2 (2.0%)	0	0
Hypertension	3 (2.9%)	1 (1.0%)	0	0
Upper respiratory tract infection	1 (1.0%)	2 (2.0%)	0	0
Infusion related reaction	2 (2.0%)	1 (1.0%)	0	0
Lung infection	0	2 (2.0%)	0	0
Febrile neutropenia	0	0	1 (1.0%)	0
Hyponatremia	0	1 (1.0%)	0	0
Abdominal infection	0	1 (1.0%)	0	0
Infection	0	2 (2.0%)	0	0
Respiratory infection	0	1 (1.0%)	0	0
Abdominal pain	0	1 (1.0%)	0	0
Allergic reaction	0	1 (1.0%)	0	0
Hyperuricemia	3 (2.9%)	0	1 (1.0%)	0
Hypokalemia	2 (2.0%)	0	1 (1.0%)	0
Heart failure	1 (1.0%)	0	1 (1.0%)	0
Autoimmune hemolytic anemia	0	0	2 (2.0%)	0
Autoimmune hepatitis	0	0	1 (1.0%)	0
Volvulus^§^	0	0	0	1 (1.0%)
Death^#^	0	0	0	1 (1.0%)
Blood bilirubin increased	1 (1.0%)	0	1 (1.0%)	0
Reticulocyte count increased	0	1 (1.0%)	0	0
Lymphopenia	1 (1.0%)	1 (1.0%)	0	0
Weight loss	2 (2.0%)	1 (1.0%)	0	0
Bone marrow hypocellular	0	0	1 (1.0%)	0
Neutropenia	0	1 (1.0%)	0	0

Data are number of patients (%). This table included all grade treatment-related adverse events occurring in at least 10% of patients and all grade 3–5 events

^§^One patient developed volvulus during treatment and resulted in death, which was deemed uncertainly related to geptanolimab treatment by investigator

^#^The death was deemed uncertainly related to treatment

^*^Treatment-related adverse events were defined as an adverse event related, possibly related or uncertainly related to treatment, as assessed by the investigator

irAE occurred in 36 (35.3%) patients with 9.8% (*n* = 10) of patients having grade 3 or above (Additional file 1: Table S6). The most common irAE was pruritus (*n* = 7, 6.9%), followed by hypothyroidism (*n* = 4, 3.9%), hyperthyroidism (*n* = 3, 2.9%), tri-iodothyronine free decreased (*n* = 3, 2.9%), tri-iodothyronine decreased (*n* = 3, 2.9%) and thyroid-stimulating hormone increased (*n* = 3, 2.9%). Of the 36 patients experiencing irAEs, the irAEs resolved without treatment or interruption of the geptanolimab in five patients. Twenty-two (21.6%) patients received treatment for their irAEs, and 12 (11.8%) of them received corticosteroids.

### Biomarker analysis

Of 94 patients with ADA samples available, two (2.1%) were tested positive for ADAs at baseline and one of them changed to be negative after geptanolimab administration. ADAs were detected in another four (4.3%) patients during treatment course. Archival FFPE tissue samples were obtained for TMB (*n* = 59) and MSI testing (*n* = 67). Most patients (*n* = 65, 97.0%) were microsatellite stable (MSS), and the median TMB was 1.2 (range 0–18.8) mutations/Mb.

**Fig. 4:**
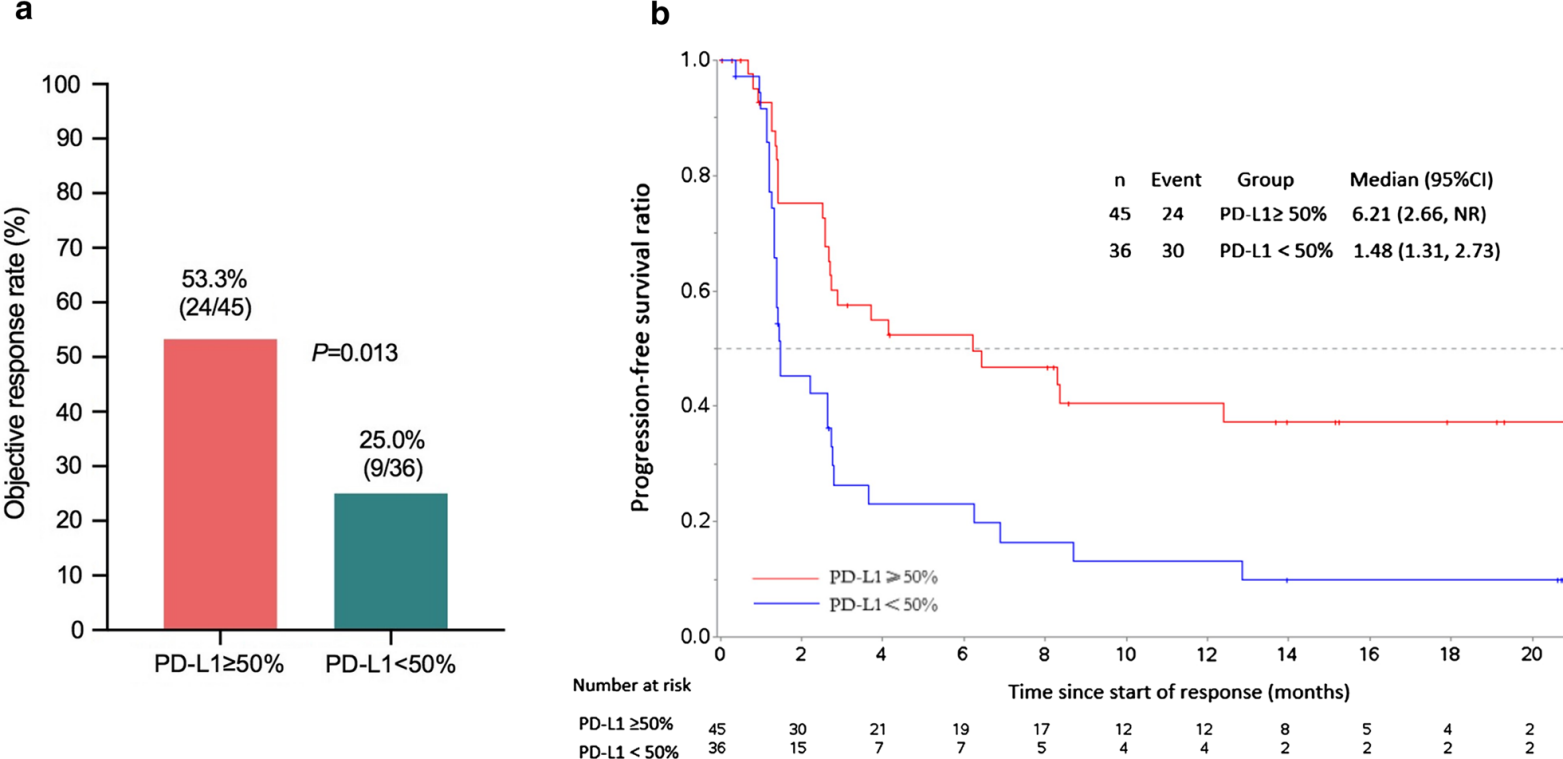
**a** Post-hoc analysis of objective response rate and **b** Kaplan–Meier estimated of progression-free survival by PD-L1 expression based on an independent radiological review committee assessment. PD-L1, programmed death-ligand 1; CI, confidence interval; NR, not reached. *Note* Responders were response-evaluable patients with PD-L1 assessed who achieved a complete or partial response as best overall response per 2014 Lugano lymphoma response criteria. PD-L1 expression was assessed with a cutoff of 50%

Collectively, 91 patients had available tissue sample for PD-L1 testing. Of the 81 patients with available PD-L1 expression data in FAS, 80 (98.8%) had PD-L1 expression of 1% or greater. More than half of patients (45/81, 55.6%) had a PD-L1 ≥ 50%, and these patients derived more benefit from geptanolimab treatment compared to < 50% ones. Post hoc analysis showed ORRs were 53.3% (24/45, 95% CI 37.9% to 68.3%) in PD-L1 ≥ 50% patients and 25.0% (9/36, 95% CI 12.1% to 42.2%) in < 50% cases (*p* = 0.013) (Fig. 4a). Additionally, PD-L1 ≥ 50% patients showed longer median PFS (6.2 months, 95% CI, 2.7 to not reached vs. 1.5 months, 95% CI 1.3 to 2.7, *p* = 0.002; Fig. 4b) and OS (not reached, 95% CI, 9.5 to not reached vs. 10.2 months, 95% CI, 6.2 to 18.5, *p* = 0.043) than PD-L1 < 50% cases.

## Discussion

Studies in r/r PTCL patients of second or later line treatment had a response rate ranging from 23 to 29% except brentuximab vedotin with a higher rate in CD30-positive PTCLs. In this study, treatment of geptanolimab demonstrated an IRRC assessed ORR of 40.4% in r/r PTCL. A subgroup analysis showed direct association of response and survival with PD-L1 ≥ 50% patients. To the best of our knowledge, this study represents the largest prospective clinical trial evaluating efficacy and safety of PD-1 inhibitor in PTCL patients.

PD-L1 expression has been approved in many indications as a determinative biomarker for conducting anti-PD-1/PD-L1 treatment. Compared to DLBCL NOS, PD-L1 expression was higher in PTCLs [30], suggesting the possibility of adopting anti-PD-1 treatment in T and NK cell malignancies.

Preclinical data reported by Wartewig T et al. suggested that the oncogenic T cell signalling upregulated PD-1 expression while PD-1 suppressed oncogenic effector pathways. Additionally, their study found that ICIs could accelerate and/or reactivate T cell clones with oncogenically activated T cell receptor pathways, thus indicated the need for special consideration of using ICIs in patients with T cell non-Hodgkin lymphoma [31].

Some results of clinical studies indicated the concern of using PD-1 antibody in T cell lymphoma patients which might accelerate the tumor progression. A phase 2 study investigating nivolumab in ATLL, an aggressive mature T cell lymphoma characterized by human T cell leukemia virus type 1 (HTLV-1), discontinued after the first three patients showed a rapid progression by the first dose [32]. Further mechanism study suggested the detrimental effects derived from the correlation of PD-1 signaling and HTLV-1 basic zipper protein. In this study, we have excluded ATLL patients to avoid the possibility of rapid progression. In addition, a previous study of nivolumab in patients with r/r PTCL, in which half of patients (6/12) were AITL, reported hyperprogressive disease in four patients [27]. Thus, we initially excluded AITL as well from enrolling in this study. Nevertheless, four patients were retrospectively confirmed AITL per central pathology review and all of them had disease control including 2 PR and 2 SD. No hyperprogressive disease was observed in these four patients. Therefore, the efficacy and safety of anti-PD-1 treatment in AITL merit further investigations.

Other clinical data in PTCLs showed some activity. A phase 2 study of 23 enrolments with nivolumab treatment in T cell lymphoma represented an ORR of 17% in which all responders achieved PR. Two patients with PTCLs had response durations of 10.6 and 78.6+ weeks [33]. One more recent phase 2 trial of pembrolizumab monotherapy in r/r mature T cell lymphoma showed an ORR of 33% (5 of 15 patients) but halted after interim futility analysis [26]. The ORR of this study was 40.4%. Moreover, among the IRRC-assessed responders, the 12-month DOR rate was 48.5%, suggesting the survival benefit of geptanolimab for r/r PTCLs. Two patients in this study recorded an increased tumor burden over 200% compared to baseline. The possibility of hyperprogression has been ruled out and determined as disease progression by the investigators.

In this study, the PD-L1 ≥ 50% rate was higher in patients with ENKTL (78.9%, 15/19) and ALCL ALK + (71.4%, 5/7), followed by ALCL ALK- (38.5%, 5/13) and PTCL-NOS (35.7%, 10/28). For B cell lymphomas with PD-L1 gene alteration and upregulation, response rate was higher with anti-PD-1 treatment [20–24]. We observed the correlation of PD-L1 expression with efficacy for PTCL patients in this study. Patients with PD-L1 expression of 50% or higher (*n* = 45) derived more benefit from geptanolimab treatment, showing an ORR of 53.3% and a median PFS of 6.2 months. Additionally, response rate was higher for patients with ENKTL, ALCL ALK- or ALCL ALK + . The associations between PD-L1 expression, pathological subtypes and response to geptanolimab need further exploration in future clinical studies of PTCLs.

The safety profile of geptanolimab is similar to previous report of geptanolimab and other anti-PD-1 antibodies with hematologic disorders seeming to be more common [26, 27, 34]. Although TRAEs including fatigue, gastrointestinal, skin disorders and pneumonitis were observed with anti-PD-1 treatment, the safety profile could vary in different malignancies [35, 36]. Patients with PTCLs are heterogenous and aggressive, likely to be treated with intensive chemotherapy previously; thus, patients with r/r PTCL were more vulnerable to treatment. In this study, the most commonly reported grade 3 or 4 TRAEs were hematological, with lymphocyte count decreased and platelet count decreased reported in seven patients, and all cases are manageable.

Limitation of this study included absence of central pathology review in other 13 patients. In addition, this study is single arm, and the follow-up period was short. Therefore, a randomized control trial with longer follow-up period is warranted to further investigate the treatment in this population.

## Conclusions

In conclusion, geptanolimab showed promising activity and manageable toxicity for patients with r/r PTCL. Given this result, anti-PD-1 antibody could be a new treatment approach for patients with r/r PTCL.

## Supplementary information

**Additional file 1:** Supplemental Methods. **Table S1** Study Site and Investigators. **Table S2** Patient baseline demographic and clinical characteristics in full analysis set (N = 89). **Table S3** Discontinuations due to Treatment-Related Adverse Events. **Table S4** Subgroup analysis per independent radiological review committee (n = 89). **Table S5** Efficacy of geptanolimab in pathological subtypes of PTCLs^&^. **Table S6** Immune related adverse events.

## Data Availability

The data that support the findings of this study are available from the corresponding author upon reasonable request.

## Abbreviations

PTCL: Peripheral T cell lymphoma
NHL: Non-Hodgkin lymphoma
PTCL-NOS: Peripheral T cell lymphoma, not otherwise specified
AITL: Angioimmunoblastic T cell lymphoma
NK: Natural killer
CHOP: Cyclophosphamide, doxorubicin, vincristine, prednisone
CHOEP: Cyclophosphamide, doxorubicin, vincristine, prednisone, etoposide
DDGP: Dexamethasone, cisplatin, gemcitabline, pegaspargase
ASCT: Autologous stem-cell transplantation
r/r: Relapsed or refractory
HDAC: Histone deacetylase
ALCL: Anaplastic large cell lymphoma
ORR: Objective response rate
DOR: Duration of response
ICI: Immune checkpoint inhibitor
PD-1: Programmed cell death protein-1
PD-L1: Programmed death-ligand 1
DLBCL: Diffuse large B cell lymphoma
ECOG: Eastern Cooperative Oncology Group
PS: Performance status
ULN: Upper limit of normal
ALT: Alanine aminotransferase
AST: Aspartate aminotransferase
ATLL: Adult T cell leukemia/lymphoma
CTLA-4: Cytolytic T lymphocyte-associated antigen-4
HIV: Human immunodeficiency virus
TP-Ab: Anti-treponema pallidum antibody
PD: Progressive disease
CT: Computed tomography
MRI: Magnetic resonance imaging
PET-CT: Positron emissions tomography and computed tomography
IRRC: Independent radiological review committee
CR: Complete response
PR: Partial response
DCR: Disease control rate
TTR: Time to response
PFS: Progression-free survival
OS: Overall survival
AE: Adverse event
TRAE: Treatment-related adverse event
irAE: Immune-related adverse event
FAS: Full analysis set
PPS: Per protocol set
CI: Confidence interval
ADA: Antidrug antibody
LBA: Ligand-binding assay
IHC: Immunohistochemistry
FFPE: Formalin-fixed paraffin-embedded
MSI: Microsatellite instability
TMB: Tumor mutation burden
ENKTL: Extranodal natural killer/T cell lymphoma, nasal type
ALK: Anaplastic lymphoma kinase
LDH: Lactate dehydrogenase
HTLV-1: Human T cell leukemia virus type 1
