# Supplementary material for: Efficacy and safety of geptanolimab (GB226) for relapsed or refractory peripheral T cell lymphoma: an open-label phase 2 study (Gxplore-002)
Source: J Hematol Oncol. 2021 Jan 12;14:12. doi: 10.1186/s13045-021-01033-1 (PMC7802130; doi:10.1186/s13045-021-01033-1)
Supplement: Supplementary file 1 — Additional file 1: Supplemental Methods. Table S1 Study Site and Investigators. Table S2 Patient baseline demographic and clinical characteristics in full analysis set (N = 89). Table S3 Discontinuations due to Treatment-Related Adverse Events. Table S4 Subgroup analysis per independent radiological review committee (n = 89). Table S5 Efficacy of geptanolimab in pathological subtypes of PTCLs&. Table S6 Immune related adverse events. [file 13045_2021_1033_MOESM1_ESM.doc]

**Supplementary File**

# Efficacy and Safety of geptanolimab (GB226) for Relapsed or Refractory Peripheral T-cell Lymphoma: an open-label phase 2 study (Gxplore-002)

Yuankai Shi, Jianqiu Wu, Zhen Wang, Liling Zhang, Zhao Wang, Mingzhi Zhang, Hong Cen, Zhigang Peng, Yufu Li, Lei Fan, Ye Guo, Liping Ma, Jie Cui, Yuhuan Gao, Haiyan Yang, Hongyu Zhang, Lin Wang, Weihua Zhang, Huilai Zhang, Liping Xie, Ming Jiang, Hui Zhou, Yuerong Shuang, Hang Su, Xiaoyan Ke, Chuan Jin, Xin Du, Xin Du, Li Liu, Yaming Xi, Zheng Ge, Ru Feng, Yang Zhang, Shengyu Zhou, Fan Xie, Qian Wang

[Supplemental Methods](#__RefHeading___Toc7651) 1

[Table S1 Study Site and Investigators](#__RefHeading___Toc7008) 2

[Table S2 Patient baseline demographic and clinical characteristics of patients in full analysis set (N=89).](#__RefHeading___Toc27931) 4

[Table S3 Discontinuations due to Treatment-Related Adverse Events](#__RefHeading___Toc4452) 6

[Table S4 Subgroup analysis per independent radiological review committee (IRRC).](#__RefHeading___Toc25799) 7

Table S5 Efficacy of geptanolimab in pathological subtypes of PTCLs 9

[Table S6 Immune Related Adverse Events.](#__RefHeading___Toc27840) 10

####

#### Supplemental Methods

#### *Tumor mutation burden (TMB)*

Library construction and sequencing

Extracted genomic DNA or FFPE DNA was amplified using four pools of primer pairs targeting coding exons of analyzed genes. Amplicons were ligated with barcoded adaptors using the Ion AmpliSeq Library Kit 2.0 (Thermo Fisher Scientific). Quality and quantity of amplified library were determined using the 2100 bioanalyzer (Agilent) and Qubit (Invitrogen). Barcoded libraries were subsequently conjugated with sequencing beads by emulsion PCR and enriched using Ion Chef system (Thermo Fisher Scientific) according to the Ion 540 Kit-Chef protocol (Thermo Fisher Scientific). Sequencing was performed on the Ion GeneStudio S5 sequencer (Thermo Fisher Scientific).

TMB analysis

Raw reads generated by the sequencer were mapped to the hg19 reference genome using the Ion Torrent Suite (version 5.10). Coverage depth was calculated using Torrent Coverage Analysis plug-in. Single nucleotide variants (SNVs) and short insertions/deletions (INDELs) were identified using the Torrent Variant Caller plug-in (version 5.10). The coverage was down-sampled to 4000. VEP (Variant Effect Predictor) (version 88) was used to annotate every variant using databases from COSMIC v.86 and Genome Aggregation database r2.0.2. Variants with coverage ≥25, allele frequency ≥ 5% and actionable variants with allele frequency ≥ 2% were retained.

This test provides uniform coverage of the targeted regions, enabling > 20 X coverage for 95% of amplicons with a mean coverage > 800x.

Variants reported in Genome Aggregation database r2.0.2 with > 1% minor allele frequency (MAF) were considered as polymorphisms. ACT Genomics in-house database was used to determine technical errors. Clinically actionable and biologically significant variants were determined based on the published medical literature.

Tumor mutational burden (TMB) was calculated by using the sequenced regions of ACTOnco®+ to estimate the number of somatic nonsynonymous mutations per megabase of all protein-coding genes (whole exome). The TMB calculation predicted somatic variants and applied a machine learning model with a cancer hotspot correction. TMB may be reported as mutations per megabase (Muts/Mb).

**Table S1** Study Site and Investigators

| **Site** | **Location** | **Principal Investigator** | **Enrolled Patients number** |
| --- | --- | --- | --- |
| Jiangsu Cancer Hospital | Nanjing, China | Jianqiu Wu | 10 |
| Linyi Cancer Hospital | Linyi, China | Zhen Wang | 9 |
| Cancer Center, Union Hospital, Tongji Medical College, Huazhong University of Science and Technology | Wuhan, China | Liling Zhang | 7 |
| Beijing Friendship Hospital, Capital Medical University | Beijing, China | Zhao Wang | 6 |
| The First Affiliated Hospital of Zhengzhou University | Zhengzhou, China | Mingzhi Zhang | 5 |
| Guangxi Medical University Affiliated Tumor Hospital & Oncology Medical College | Nanning, China | Hong Cen | 5 |
| The First Affiliated Hospital of Guangxi Medical University | Nanning, China | Zhigang Peng | 5 |
| Henan Cancer Hospital | Zhengzhou, China | Yufu Li | 4 |
| Jiangsu Province Hospital | Nanjing, China | Lei Fan | 4 |
| Shanghai East Hospital | Shanghai, China | Ye Guo | 4 |
| Sun Yat-Sen Memorial Hospital and Sun Yat-Sen University | Guangzhou, China | Liping Ma | 4 |
| Gansu Provincial Cancer Hospital | Lanzhou, China | Jie Cui | 4 |
| The Fourth Hospital of Hebei Medical University | Shijiazhuang, China | Yuhuan Gao | 3 |
| Zhejiang Cancer Hospital | Hangzhou, China | Haiyan Yang | 3 |
| The Fifth Affiliated Hospital Sun Yat-Sen University | Zhuhai, China | Hongyu Zhang | 3 |
| Hainan General Hospital | Haikou, China | Lin Wang | 3 |
| The First Affiliated Hospital of Shanxi Medical University | Taiyuan, China | Weihua Zhang | 2 |
| Tianjin Medical University Cancer Institute & Hospital | Tianjin, China | Huilai Zhang | 2 |
| West China Hospital, Sichuan University | Chengdu, China | Liping Xie | 2 |
| West China Hospital, Sichuan University | Chengdu, China | Ming Jiang | 2 |
| Hunan Cancer Hospital | Changsha, China | Hui Zhou | 2 |
| Jiangxi Cancer Hospital | Nanchang, China | Yuerong Shuang | 2 |
| The Fifth Medical Center of PLA General Hospital | Beijing, China | Hang Su | 2 |
| Peking University Third Hospital | Beijing, China | Xiaoyan Ke | 1 |
| Cancer Center of Guangzhou Medical University | Guangzhou, China | Chuan Jin | 1 |
| Guangdong Provincial People’s Hospital, Guangdong Academy of Medical Sciences | Guangzhou, China | Xin Du | 1 |
| Shenzhen Second People's Hospital | Shenzhen, China | Xin Du | 1 |
| Tangdu Hospital of the Fourth Military Medical University | Xi’an, China | Li Liu | 1 |
| The First Hospital of Lanzhou University | Lanzhou, China | Yaming Xi | 1 |
| Zhongda Hospital Southeast University | Nanjing, China | Zheng Ge | 1 |
| Nanfang Hospital, Southern Medical University | Guangzhou, China | Ru Feng | 1 |
| The Second Hospital of Dalian Medical University | Dalian, China | Yang Zhang | 1 |
| Cancer Hospital, Chinese Academy of Medical Sciences & Peking Union Medical College | Beijing, China | Yuankai Shi | 0 |

**Table S2** Patient baseline demographic and clinical characteristics in full analysis set (N=89).

| Patient characteristics | N (%) |
| --- | --- |
| Age |  |
| Median, years (range) | 52.0 (18 -78) |
| <65 | 75 (84.3) |
| ≥65 | 14 (15.7) |
| Gender, n (%) |  |
| Male | 60 (67.4) |
| Female | 29 (32.6) |
| ECOG PS |  |
| 0 | 15 (16.9) |
| 1 | 74 (83.1) |
| Prior lines of systemic therapy |  |
| 1 | 37 (41.6) |
| 2 | 28 (31.5) |
| 3 or above | 24 (27.0) |
| Stage of disease |  |
| I-II | 17 (19.1) |
| III-IV | 72 (80.9) |
| Pathological subtype^$^ |  |
| PTCL-NOS | 28 (31.5) |
| Extranodal NK/T-cell lymphoma, nasal type | 19 (21.3) |
| ALCL ALK- | 13 (14.6) |
| ALCL ALK+ | 7 (7.9) |
| Other subtypes * | 22 (24.7) |
| Prior therapies |  |
| Multiagent regimen |  |
| For ENKTL |  |
| Asparaginase-based chemotherapy^†^ | 17 (89.5) |
| For other subtype of PTCL |  |
| Anthracycline-containing chemotherapy^‡^ | 66 (94.3) |
| Single-agent regimen |  |
| Chidamide | 20 (22.5) |
| Gemcitabine | 48 (53.9) |
| Methotrexate | 10 (11.2) |
| Bortezomib | 1 (1.1) |
| Radiotherapy | 30 (33.7) |
| Autologous stem-cell transplantation | 5 (5.6) |

^$^ Histologically diagnosis per central pathology review.

^*^ Other subtypes include: 8 cases of unclassifiable PTCL, 2 cases of Type II enteropathy-associated T-cell lymphoma (EATL, type II), 4 cases of AITL ( diagnosed as other PTCL subtypes by study site pathologist [one PTCL, others and three PTCL-NOS] and classified later on as AITL per central pathology review), and 8 cases of cutaneous T Cell lymphoma, including 3 cases of mycosis fungoides (MF), 1 case of skin γ σ cutaneous T-cell lymphomas (γ σ CTCL), 2 case of primary cutaneous ALCL and 2 cases of primary cutaneous PTCL-NOS.

^†^ The proportion (%) was defined as the number of patients receiving asparaginase-based chemotherapy divided by the number of those diagnosed with ENKTL.

^‡^ The proportion (%) was defined as the number of patients receiving anthracycline-containing chemotherapy divided by the number of those diagnosed with other subtypes of PTCL except for ENKTL.

Abbreviations: ENKTL, extranodal NK/T-cell lymphoma, nasal type; ALCL, anaplastic large-cell lymphoma; ALK, anaplastic lymphoma kinase; CHOP, cyclophosphamide, doxorubicin, vincristine, and prednisone; ECOG, Eastern Cooperative Oncology Group; PS, performance status; NK, natural killer; PTCL, peripheral T-cell lymphoma; NOS, not otherwise specified; AITL, angioimmunoblastic T-cell lymphoma.

**Table S3** Discontinuations due to Treatment-Related Adverse Events.

| Patient Age (years/sex) | Event | Grade | Study Day |
| --- | --- | --- | --- |
| 49/male | Pneumonitis | 4 | D209 |
| 42/male | Autoimmune hepatitis | 4 | D27 |
| 78/male | Autoimmune hemolytic anemia | 4 | D73 |
| 63/male | Death | 5 | D337 |
| 58/female | Platelet count decreased | 4 | D8 |
| 47/female | Autoimmune hemolytic anemia | 4 | D400 |
| 53/female | Pneumonitis | 2 | D72 |
| 63/male | Febrile neutropenia | 4 | D87 |
| 61/male | Platelet count decreased | 4 | D7 |
| 58/male | Nephritis | 2 | D54 |
| 62/male | Pruritus | 3 | D420 |

**Table S4** Subgroup analysis per independent radiological review committee (n=89).

| Characteristic | No. of patients | No. of responders | ORR**^§^** (%)  (95%CI) | p | median PFS (95%CI) mo | p |
| --- | --- | --- | --- | --- | --- | --- |
| Age |  |  |  |  |  |  |
| <65 | 75 | 32 | 42.7  (31.3 to 54.6) | 0.386 | 2.8 (2.6 to 6.2) | 0.035 |
| ≥65 | 14 | 4 | 28.6  (8.4 to 58.1) |  | 1.4 (1.3 to 4.2) |  |
| Prior lines of systemic therapy |  |  |  |  |  |  |
| 1 | 37 | 18 | 48.6  (31.9 to 65.6) | 0.197 | 8.4  (2.7 to NR) | 0.001 |
| ≥2 | 52 | 18 | 34.6  (22.0 to 49.1) |  | 2.5 (1.4 to 2.9) |  |
| Elevated LDH level**^#^** |  |  |  | 0.081 |  | 0.073 |
| No | 51 | 25 | 49.0  (34.8 to 63.4) |  | 4.2  (2.6 to 8.4) |  |
| Yes | 38 | 11 | 28.9  (15.4 to 45.9) |  | 1.5  (1.3 to 2.8) |  |
| Previous exposure to Chidamide |  |  |  | 0.313 |  | 0.246 |
| Yes | 20 | 6 | 30.0  (11.9 to 54.3) |  | 2.2 (1.2 to 6.2) |  |
| No | 69 | 30 | 43.5  (31.6 to 56.0) |  | 2.8  (2.6 to 6.2) |  |
| Prior ASCT |  |  |  | 0.153 |  | 0.157 |
| Yes | 5 | 4 | 80.0  (28.4 to 99.5) |  | NR  (0.8 to NR) |  |
| No | 84 | 32 | 38.1  (27.7 to 49.3) |  | 2.7  (1.5 to 4.2) |  |
| Baseline bone marrow involvement |  |  |  |  |  |  |
| Yes | 11 | 3 | 27.3  (6.0 to 61.0) | 0.514 | 2.2 (0.8 to 6.2) | 0.201 |
| No | 78 | 33 | 42.3  (31.2 to 54.0) |  | 2.8 (2.5 to 6.2) |  |

^#^ LDH level were obtained before enrolment.

^§^Best overall response by independent radiological review committee review before the data cutoff.

Abbreviations: ORR, objective response rate; PFS, progression-free survival; LDH, lactate dehydrogenase; CI, confidence interval; NR, not reached; ASCT, autologous hematopoietic stem cell transplantation.

**Table S5** Efficacy of geptanolimab in pathological subtypes of PTCLs^&^.

| Pathological subtype | ORR | | DCR | |
| --- | --- | --- | --- | --- |
|  | N | % | N | % |
| PTCL-NOS (n=28) | 5 | 17.9 | 13 | 46.4 |
| Extranodal NK/T-cell lymphoma, nasal type (n=19) | 12 | 63.2 | 14 | 73.7 |
| ALCL ALK- (n=13) | 7 | 53.8 | 8 | 61.5 |
| ALCL ALK+ (n=7) | 3 | 42.9 | 5 | 71.4 |
| PTCL, others (n=8) | 4 | 50.0 | 6 | 75.0 |
| AITL (n=4) | 2 | 50.0 | 4 | 100.0 |
| Type II enteropathy-associated T-cell lymphoma (n=2) | 0 | 0 | 0 | 0 |
| Cutaneous T cell lymphoma (n=8) |  |  |  |  |
| Mycosis fungoides (n=3) | 0 | 0 | 0 | 0 |
| Skin γ σ cutaneous T-cell lymphomas (n=1) | 0 | 0 | 0 | 0 |
| Primary cutaneous ALCL (n=2) | 2 | 100 | 2 | 100 |
| Primary cutaneous PTCL-NOS (n=2) | 1 | 50 | 1 | 50.0 |

^&^ Eficacy analysis was done in full analysis set (N=89) per independent radiological review committee review assessment.

^§^Best overall response by independent radiological review committee review before the data cutoff.

Abbreviations: PTCL, peripheral T-cell lymphoma; ORR, overall response rate; DCR, disease control rate; NOS, not otherwise specified; AITL, angioimmunoblastic T-cell lymphoma; ALCL, anaplastic large-cell lymphoma; ALK, anaplastic lymphoma kinase; NK, natural killer.

**Table S6** Immune related adverse events.

|  | **Immune Related Adverse Event (n=102)** | | |
| --- | --- | --- | --- |
|  | **Grade 1-2** | **Grade 3** | **Grade 4** |
| White blood cell count decreased | 2 (2.0%) | 0 | 0 |
| Blood thyroid stimulating hormone increased | 3 (2.9%) | 0 | 0 |
| Hypothyroidism | 4 (3.9%) | 0 | 0 |
| Hyperthyroidism | 3 (2.9%) | 0 | 0 |
| Respiratory tract infection | 0 | 1 (1.0%) | 0 |
| Lung Infection | 0 | 2 (2.0%) | 0 |
| Pneumonitis | 1 (1.0%) | 0 | 1 (1.0%) |
| Tri-iodothyronine decreased | 3 (2.9%) | 0 | 0 |
| Rash | 1 (1.0%) | 1 (1.0%) | 0 |
| Pruritus | 6 (5.9%) | 1 (1.0%) | 0 |
| Thyroxine decreased | 2 (2.0%) | 0 | 0 |
| Tri-iodothyronine free decreased | 3 (2.9%) | 0 | 0 |
| Alanine aminotransferase increased | 2(2.0%) | 0 | 0 |
| Autoimmune hepatitis | 0 | 0 | 1 (1.0%) |
| Autoimmune hemolytic anemia | 0 | 0 | 1 (1.0%) |
| Platelet count decreased | 0 | 0 | 1(1.0%) |
| Allergic reaction | 0 | 1 (1.0%) | 0 |
| Infusion related reaction | 1 (1.0%) | 1 (1.0%) | 0 |
| Fever | 2 (2.0%) | 0 | 0 |
| Upper respiratory infection | 0 | 1 (1.0%) | 0 |
| Febrile neutropenia | 0 | 0 | 1 (1.0%) |
| Arthralgia | 2 (2.0%) | 0 | 0 |

Data are number of patients (%). This table included all grade treatment-related adverse events occurring in at least 2% of patients and all grade 3-5 events.
